# NADPH Oxidase Gene, *FgNoxD*, Plays a Critical Role in Development and Virulence in *Fusarium graminearum*

**DOI:** 10.3389/fmicb.2022.822682

**Published:** 2022-03-03

**Authors:** Taiying Li, Dohyun Kim, Jungkwan Lee

**Affiliations:** Department of Applied Biology, Dong-A University, Busan, South Korea

**Keywords:** *Fusarium graminearum*, NADPH oxidase, virulence, sexual development, stress

## Abstract

NADPH oxidase is an enzyme that generates reactive oxygen species from oxygen and NADPH and is highly conserved in eukaryotes. In *Fusarium graminearum*, a series of different Nox enzymes have been identified. NoxA is involved in sexual development and ascospore production and, like NoxB, also contributes to pathogenicity. Both NoxA and NoxB are regulated by the subunit NoxR, whereas NoxC is usually self-regulated by EF-hand motifs found on the enzyme. In this study, we characterized another NADPH oxidase in *F. graminearum*, *FgNoxD*. In the *FgNoxD* deletion mutant, vegetative growth and conidia production were reduced, while sexual development was totally abolished. The *FgNoxD* deletion mutant also showed reduced resistance to cell wall perturbing agents; cell membrane inhibitors; and osmotic, fungicide, cold, and extracellular oxidative stress, when compared to the wild type. Moreover, in comparison to the wild type, the *FgNoxD* deletion mutant exhibited reduced virulence against the host plant. The *FgNoxD* deletion mutant produced less deoxynivalenol than the wild type, and the *Tri5* and *Tri6* gene expression was also downregulated. In conclusion, our findings show that *FgNoxD* is involved in the survival against various stresses, conidiation, sexual development, and virulence, highlighting this enzyme as a new target to control the disease caused by *F. graminearum*.

## Introduction

*Fusarium graminearum* is a homothallic ascomycete fungus that causes *Fusarium* head blight (FHB) in cereal crops worldwide (Leslie and Summerell, 2006). It produces deoxynivalenol (DON) which inhibits protein synthesis by binding to ribosomes, making it toxic to humans and animals (Desjardins and Proctor, 2007; Pestka, 2010; Chong et al., 2020). Despite the major economic and health impacts caused by *F. graminearum*, sufficient strategies to control FHB have not been developed.

Reactive oxygen species (ROS) are highly reactive chemical molecules that play important roles in cell signaling, cell growth, and homeostasis (Dunand et al., 2007; Veal and Day, 2011). ROS are generated by all aerobic organisms as byproducts of normal metabolism. Excess ROS, such as superoxide, hydrogen peroxide (H_2_O_2_), and hydroxyl radicals, can non-specifically and rapidly react with other molecules including lipids, proteins, DNA, and carbohydrates (Gutteridge, 1994; Rodriguez and Redman, 2005). These reactions cause DNA mutation, lipid peroxidation, and protein oxidation, resulting in cellular dysfunction and apoptosis (Aguirre et al., 2005; Halliwell and Gutteridge, 2015).

NADPH oxidases (Nox), a ROS-producing enzyme, is membrane-bound enzyme complex exposed to the extracellular space. This multicomponent Nox enzyme complex was first studied in human phagocytic cells, where it was found that NADPH is used as an electron donor and the electrons are transported through the membrane to convert oxygen to superoxide (Lambeth, 2004). In animal cells, Nox enzymes are linked to cell signaling, cell growth, and cell death (Sumimoto, 2008; Brown and Griendling, 2009). In plant cells, Nox is implicated in the response to abiotic stresses, infection by pathogens, and polarized growth of root hairs. Additionally, Nox also acts as a secondary messenger for speedy transmission over long distance and in local signaling (Torres et al., 2002; Foreman et al., 2003; Suzuki et al., 2011; Lee et al., 2020).

Nox enzyme have also been studied in fungi. Four different fungal Nox enzymes—NoxA (Nox1), NoxB (Nox2), NoxC (Nox3), and NoxD—have been characterized to date. The gh91^phox^ protein homolog enzyme, NoxA, is involved in fruiting body formation in various filamentous fungi, including *Aspergillus nidulans*, *Podospora anserina*, and *Neurospora crassa* (Lara-Ortíz et al., 2003; Malagnac et al., 2004; Cano-Dominguez et al., 2008). Furthermore, NoxA is also related to virulence, formation of sclerotia, and cellulose degradation (Giesbert et al., 2008; Segmuller et al., 2008; Brun et al., 2009; Kim et al., 2011; Yang and Chung, 2012). Another gh91^phox^ protein homolog enzyme, NoxB, is necessary for host penetration in *Magnaporthe oryzae* and *Botrytis cinerea*, and ascospore germination in *N. crassa* and *P. anserina* (Malagnac et al., 2004; Egan et al., 2007; Cano-Dominguez et al., 2008; Segmuller et al., 2008). Although many fungi, for example, *M. oryzae* and *P. anserine*, express the NoxC enzyme, little is known about this enzyme and its function, except for its regulatory subunits (Takemoto et al., 2007).

In phagocytes, gh91^phox^ binds to the p22^phox^ protein, both of which are subunits of flavocytochrome b_558_, necessary for activation of the Nox enzyme. Moreover, the transmembrane protein also applies to Nox1, Nox3, and Nox4 (Nakano et al., 2008; Zana et al., 2018; Makhezer et al., 2019). In fungi, the functional orthologue of the p22^phox^ protein was first identified in *Sordaria macrospora* which was named Pro41. In *S. macrospora*, Pro41 is required for fruiting body maturation (Nowrousian et al., 2007, 2012; Galhano et al., 2017). The membrane protein NoxD is highly homologous to the endoplasmic reticulum (ER) protein Pro41 in several fungi (Nowrousian et al., 2007; Lacaze et al., 2015; Siegmund et al., 2015). In *B. cinerea*, NoxA and NoxD interact with each other and are involved in pathogenicity, fusion of conidial anastomosis tube, and formation of sclerotia and conidia (Siegmund et al., 2015).

*Fusarium graminearum* also expresses NoxA, NoxB, NoxC, and NoxD. Study of NoxA and NoxB in *F. graminearum* indicate that NoxA is involved in perithecia development and ascospore production, and both NoxA and NoxB contribute to virulence but are not associated with mycotoxin synthesis (Wang et al., 2014). NoxC in *F. graminearum* is typically self-regulated by EF-hand motifs found on the enzyme, whereas NoxA and NoxB are regulated by the regulatory subunit NoxR (Heller and Tudzynski, 2011; Tudzynski et al., 2012; Zhang et al., 2016). Although the Pro41 homolog gene NoxD is also found in *F. graminearum*, the function of the gene product has not yet been studied. Therefore, in this study, we identified the location and characterized the functions of *NoxD* in *F. graminearum* (*FgNoxD* for *F. graminearum NoxD*). The study determined the phenotypic changes in conidial germination, vegetative growth, virulence, and mycotoxin synthesis to determine the biological functions of *FgNoxD*.

## Materials and Methods

### Fungal Strains and Culture Media

*Fusarium graminearum* wild-type strain GZ3639 (Bowden and Leslie, 1999) and mutants were cultivated in media following the *Fusarium* laboratory manual (Leslie and Summerell, 2006). Conidia formation was induced in carboxyl methyl cellulose (CMC; Cappellini and Peterson, 1965) or yeast malt agar (YMA) medium (Harris, 2005), and fungal strains were cultivated in complete medium (CM). All strains were stored as agar block in 20% glycerol at −80°C.

### Transformation

Targeted gene deletion and complementation were manipulated according to the split-marker recombination (SMR) strategy (Catlett et al., 2003). For gene deletion, the 5' and 3' flankings of the target gene were amplified from GZ3639. Meanwhile, a hygromycin resistance cassette (HYG) was amplified from pIGPAPA (Horwitz et al., 1999) using primer pairs. The three amplicons were mixed and fused using PCR. The final product for transformation was amplified during the third PCR step using nested primer pairs. To complement the gene deletion, a DNA fragment carrying the open read frame and native promoter of *FgNoxD* was fused with the geneticin resistance cassette (GEN) and amplified with pII99 through SMR (Namiki et al., 2001). For transformation, protoplasts of GZ3639 were prepared and a previously described method was applied (Kim et al., 2006; Li et al., 2019). In brief, conidia were incubated in 50 ml of YPG (10 g/L of peptone, 3 g/L of yeast extract, and 20 g/L of glucose) with shaking at 200 rpm for 12 h at 25°C. After that, the mycelia were harvested by filtration, then incubated in 35 ml of 1 M NH_4_Cl containing 15 mg/ml driselase (Sigma-Aldrich) to generate protoplast. The final PCR product, which carried a selectable marker, was incorporated directly into the protoplast. Transformants carrying selectable markers were selected on regeneration medium (1 g/L of casein, 1 g/L of yeast extract, 342 g/L of sucrose, and 15 g/L of micro agar) containing 75 μg/ml hygromycin or 75 μg/ml geneticin. The PCR primers used in this study are listed in Table 1.

**Table 1:** Primers used for genetic manipulation in this study.

Primer name	Sequence (5' → 3')
***FgNoxD* deletion mutant**	
5'F primer	AGTCAACCAACACCAGATCTGCC
5'F nested primer	GTGGGCGGGAGGGAAAACC
5'R primer	TGTAAGTGGCATGGAGGGAAGC
HYG F primer	GGCTTGGCTGGAGCTAGTGGAGG
HYG R primer	TAACTGGTTCCCGGTCG
3'F primer	CTGGACGTTGTTTGGCTGTTTACC
3'R nested primer	AGTTCCCCGAGCGCCAGG
3'R primer	AGACAAGGAGCCCAGGGAACACT
HYG nestedF	GATGTAGGAGGGCGTGGATATGT
HYG nestedR	GAACCCGCTCGTCTGGCTAAGA
***FgNoxD* complemented strain**	
5'F primer	CTACCCGCCCATGCTTCT
5'F nested primer	CGAGGTCAACACCAATTACCA
5'R primer	TGCACGAGATTGTCCGCC
GEN F primer	TTATCTTTGCGAACCCAGGG
GEN R primer	CGACAGAAGATGATATTGAAGG
3'F primer	CTGGACGTTGTTTGGCTGTTT
3'R primer	GGCATATTTGATGATAGCGCC
3'R nested primer	GCCACACAAGTGGACACC
5'R nested primer	TCTCCTGTCATCTCACCTTG
3'F nested primer	TCCTGAACACCATTTGTCTCAAC
***FgNoxD* qRT-PCR**	
*FgNoxD*_F	GGCTGCCATCGAGTGCTTCTTC
*FgNoxD*_R	AACCAGCGACGAAATTAAGAGGCC
***Tri5* qRT-PCR**	
*Tri5*_F	GACCCTAAGCGACTACAG
*Tri5*_R	GTGCTACGGATAAGGTTC
***Tri6* qRT-PCR**	
*Tri6*_F	AGCGCCTTGCCCCTCTTTG
*Tri6*_R	AGCCTTTGGTGCCGACTTCTTG
***CYP* qRT-PCR**	
*CYP*_F	TCAAGCTCAAGCACACCAAGAAGG
*CYP*_R	GGTCCGCCGCTCCAGTCT

### Quantitative Real-Time PCR

To validate the constructed mutant and differently expressed genes involved in DON production, quantitative real-time PCR (qRT-PCR) was performed. The conidia of each strain (1 × 10^5^ conidia/ml) were cultivated in 20 ml of CM at 200 rpm and 25°C for 3 days. Mycelia were harvested and ground using liquid nitrogen before the total RNA of each strain was extracted using the easy-spin Total RNA Extraction Kit (iNtRON Biotechnology, Seongnam, Korea) following the manufacturer’s protocol. Next, cDNA was generated using the First Strand cDNA Synthesis Kit (TOYOBO Co., Osaka, Japan) following the manufacturer’s instructions. The synthesized cDNA of each strain was diluted to 100 ng/μl and 2 μl of cDNA was used for qRT-PCR. The qRT-PCR conditions were 95°C for 5 min, followed by 40 cycles of 95°C for 5 s, 60°C for 10 s, and 72°C for 35 s. Relative gene expression was normalized to that of cyclophilin (*CYP*; Son et al., 2013).

### Mycelia Growth and Conidia Germination

The GZ3639, *FgNoxD* deletion mutant (ΔFgNoxD), and complementation (FgNoxD-C) strains were cultivated on potato dextrose agar (PDA), CM, minimal medium (MM), and YMA for 3 days at 25°C, after which the colony diameter of each strain was measured. The aerial mycelia growth of each strain was measured as previously described, with slight modifications (Nguyen et al., 2011). Briefly, each strain was inoculated in CM in a test tube at 25°C for 3 days and aerial mycelial growth was measured. For conidial germination of each strain, conidia (1 × 10^5^ conidia/ml) harvested from CMC were incubated in MM. The number of total conidia and germinated conidia were counted at 4, 8, and 12 h by light microscopy.

### Perithecia Development

Perithecial production was induced as described in a previous study (Min et al., 2010). Each strain was inoculated onto carrot agar medium for 8 days at 25°C in the dark. Thereafter, the mycelia were removed with 1 ml of 2.5% Tween-20 and the plates were incubated under near-ultraviolet light (20 W, 50 lux) for 10 days at 25°C. Perithecia and ascospores were observed and photographed using Moticam Pro S5 Lite camera (Motic, Barcelona, Spain).

### Stress Tests

The role of *FgNoxD* in stress response was tested as described in previous studies with slight modifications. For the osmotic stress test, each strain was inoculated in MM supplemented with 1.4 M KCl and NaCl and cultivated for 5 days at 25°C, after which radial growth was measured (Gu et al., 2015). Cold stress tests were performed as previously described (Li et al., 2019). Briefly, conidia (1 × 10^3^ conidia/ml) in distilled water were stored at 4°C for 6 days and 100 μl of each suspension was spread on PDA. The number of surviving spores was counted after 1 day. The cell wall and membrane integrity of each stain were tested on MM supplemented with 60 mg/L Congo red (CR; Sigma-Aldrich), 50 μg/ml calcofluor white (CFW; Sigma-Aldrich), and 0.01% of SDS. The colony diameter of each strain was measured after cultivation at 25°C for 3 days (Ram and Klis, 2006; Schroeder and Ikui, 2019). For the fungicide resistance test, mycelia from each strain were inoculated onto MM supplemented with 0.1–0.5 mg/L prochloraz fungicide and cultivated at 25°C for 5 days (Li et al., 2019). Oxidative stress tests were performed using menadione (Katikireddy et al., 2018; Majiene et al., 2019; Funk et al., 2021) and H_2_O_2_. Each strain was inoculated in MM supplemented with 1 or 3 mM menadione and cultivated at 25°C for 3 days. Different concentrations (1, 3, and 5 mM) of H_2_O_2_ were added to MM and mycelia plugs of each strain were cultivated at 25°C for 1 day. Then mycelia plug were transmitted to a new CM and cultivated for another 3 days at 25°C to investigate the survival of each strain.

### Lipid Body Staining

Lipid body staining was performed after treatment with cold stress. Briefly, conidia (1 × 10^6^ conidia/ml) in distilled water were stored at 4°C for 1 day. The conidia were harvested by centrifugation and washed twice with phosphate-buffered saline (PBS). Thereafter, the lipid body in each strain was stained with a Nile Red solution consisting of 0.01 mg/ml Nile Red Oxazone (Sigma-Aldrich; Seong et al., 2008; Jung et al., 2018). The samples were incubated for 15 min at room temperature and washed twice with PBS. Fluorescence emitted by the lipid body was observed using Olympus BX50 microscope (Olympus, Tokyo, Japan).

### Virulence Test and DON Production

Virulence of each strain was evaluated using the wheat cultivar, Geumgangmil, and the rice cultivar DongjinByeo. For the virulence tests on wheat, plant at two different stages were inoculated. Before inoculating each strain on coleoptile, wheat seeds were germinated on moist filter paper at 25°C. Then top 2–3 mm of the coleoptiles were removed and 2 μl of conidia suspension (1 × 10^6^ conidia/ml in 0.01% Tween-20) was inoculated. The coleoptiles were then cultivated in a growth chamber at 25°C with 100% relative humidity and 12 h of light per 24 h. The virulence of each strain was assessed by measuring the length of the lesion on the diseased stem 10 days after inoculation (Wu et al., 2005). For wheat head inoculation, 10 μl of conidia suspension (1 × 10^6^ conidia/ml in 0.01% Tween-20) was inoculated into the center of each spikelet. Spikelet exhibiting FHB symptoms were counted 14 days after inoculation. The rachis of each wheat head were also examined (Lee et al., 2009). For rice head inoculation, rice heads were dipped into suspensions of each strain (1 × 10^5^ conidia/ml in 0.01% Tween-20) for 30 s and individually sealed in plastic bags for 72 h. The infected rice heads were then placed in a greenhouse and rice exhibiting FHB symptoms were counted after inoculation (Jung et al., 2018).

The production of DON was evaluated as described in a previous study (Ponts et al., 2006). Briefly, conidia of each strain were cultivated in 20 ml of GYEP medium (10 g/L glucose, 1 g/L yeast extract, and 1 g/L peptone) supplemented with or without 1 mM of H_2_O_2_ for 5 days at 200 rpm and 25°C. DON concentrations were determined using an enzyme-linked immunosorbent assay kit (CUSABIO, College Park, MD, United States) following the manufacturer’s instructions (Yoshizawa et al., 2004; Jung et al., 2018; Xu et al., 2018). Furthermore, mycelia were cultured with same method to detect the transcript levels of *Tri5* (Maier et al., 2006) and *Tri6* (Nasmith et al., 2011) genes.

### Statistical Analysis

Statistical differences of mycelial growth, cold stress, cell wall, and membrane stress tests, and virulence were examined by parametric one-way analysis of variance using R software (version 4.0.2). Additionally, statistical differences in osmotic stress resistance, DON production, and qRT-PCR were examined using *t*-test.

## Results

### Transformation and Phylogenetic Analysis of FgNoxD

**Figure 1:**
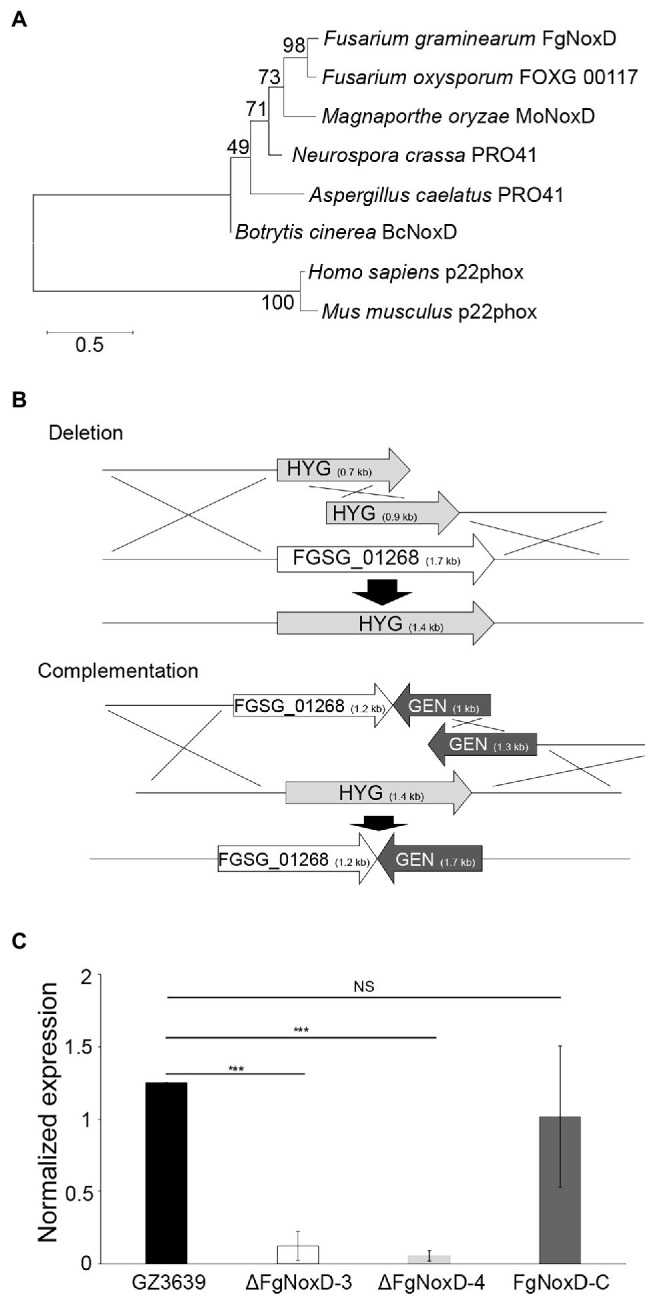
FgNoxD amino sequence phylogenetic tree and gene manipulation. **(A)** Phylogenetic analysis of FgNoxD protein. All amino acid sequences were aligned using ClustalW which built-in MEGA X (version 6.0). The phylogenetic tree analyses were performed using the method of Maximum likelihood (ML). **(B)** Homologous recombination for construction of *FgNoxD* deletion and complementation constructs. Complementation strain was created using the ΔFgNoxD-3. **(C)** Relative expression levels of *FgNoxD* in GZ3639, ΔFgNoxD, and FgNoxD-C. Error bars indicate standard errors from four repeated experiments with three biological replications. Asterisks indicate value of *p* (NS, no significance; ^***^p < 0.001) after comparison with Welch’s *t*-test. HYG, hygromycin resistance cassette and GEN, geneticin resistance cassette.

The gene sequence of *FgNoxD* (*FGSG_01268*) was acquired from National Center for Biotechnology Information database^1^ using the BcNoxD protein sequence of *B. cinerea*. *FgNoxD*, which contains 1709 bp with two introns, is predicted to encode a protein with 146 amino acids. Phylogenetic analysis and protein alignment indicated that NoxD was highly conserved in eukaryote (Figure 1A; Supplementary Figure S1).

To characterize the functions of *FgNoxD*, the *FgNoxD* gene was replaced with a constitutively expressed HYG cassette *via* SMR (ΔFgNoxD). To verify whether the observed changes found in the deletion mutant were caused by gene defection, *FgNoxD* was reintroduced at an alternate site in the deletion mutant (FgNoxD-C; Figure 1B). qRT-PCR showed that the transcripts of *FgNoxD* were completely abolished in the deletion mutant but was recovered in FgNoxD-C (Figure 1C).

### Effects of *FgNoxD* on Normal Mycelia Growth, Conidia Production, and Sexual Development

**Figure 2:**
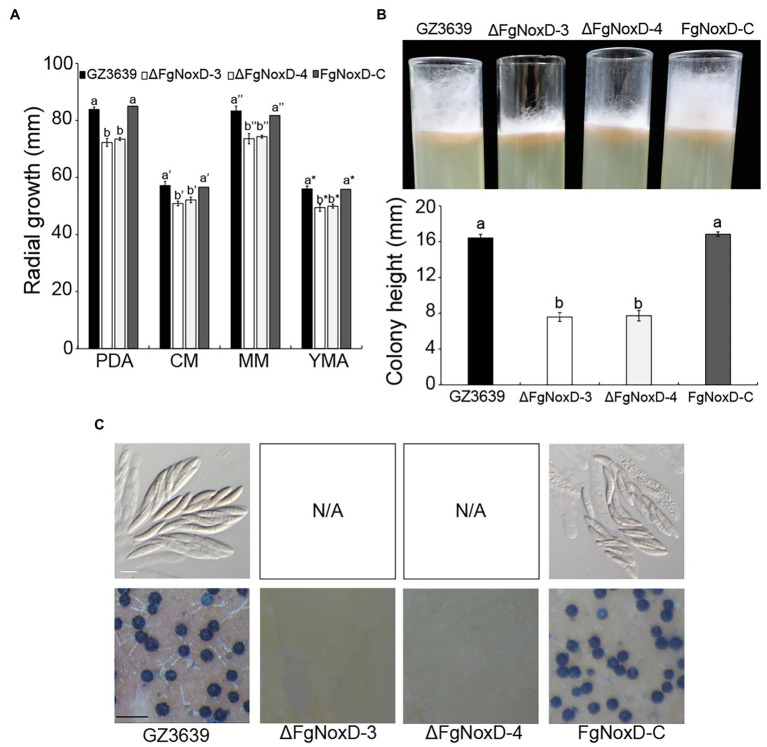
Fungal growth and sexual development GZ3639, ΔFgNoxD, and FgNoxD-C. **(A)** The growth of GZ3639, ΔFgNoxD-3, ΔFgNoxD-4, and FgNoxD-C on potato dextrose agar (PDA), complete medium (CM), minimal medium (MM), yeast malt agar (YMA) for 3 days at 25°C. Colony diameters of the strains were measured. Error bars represent the SD from five replicates. **(B)** Aerial hyphae growth of the strains on CM for 3 days. Colony height of the strains was measured. Error bars represent the SD from three replicates. **(C)** Sexual development induced on carrot agar (CA) for 10 days at near UV. Perithecia formation and ascospores were observed after 10 days. White bar = 10 μm; Black bar = 1 mm. Values with different letters are significantly different according to Tukey’s test ( *p* < 0.001).

Compared to GZ3639 and FgNoxD-C, ΔFgNoxD showed significantly reduced mycelia growth and aerial hyphae growth (Figures 2A,B). Although the conidial germination and morphology were not significantly different between GZ3639 and ΔFgNoxD, conidia production was significantly reduced in ΔFgNoxD (Table 2). Moreover, ΔFgNoxD completely lost self-fertility and did not form any initial perithecia structures (Figure 2C).

**Table 2:** Asexual development and conidia production.

Strain	Germination rate (%)^a^	Conidia morphology^b^			Conidia production (No./ml)^c^
		Length (μm)	Width (μm)	No. of septa	
GZ3639	38 A^d^	51 A	7.0 A	4.5 A	2.1 × 10^6^ A
ΔFgNoxD-3	39 A	50 A	7.1 A	4.1 A	6.9 × 10^5^ B
ΔFgNoxD-4	40 A	50 A	7.0 A	4.2 A	5.7 × 10^5^ B
FgNoxD-C	39 A	49 AC	7.1 A	4.3 A	1.5 × 10^6^ AB

a *Germination rate measured 8 h after inoculation in MM broth medium*.

b *Thirty conidia harvested from YMA for each strain were observed by microscopy*.

c *Produced conidia were evaluated by counting the number of conidia produced in carboxyl methyl cellulose medium*.

d *Values within a column with different letters are significantly different (*p* < 0.05) based on Tukey test*.

### Effects of *FgNoxD* on Cell Wall and Membrane Integrity and Oxidative Stress Resistance

**Figure 3:**
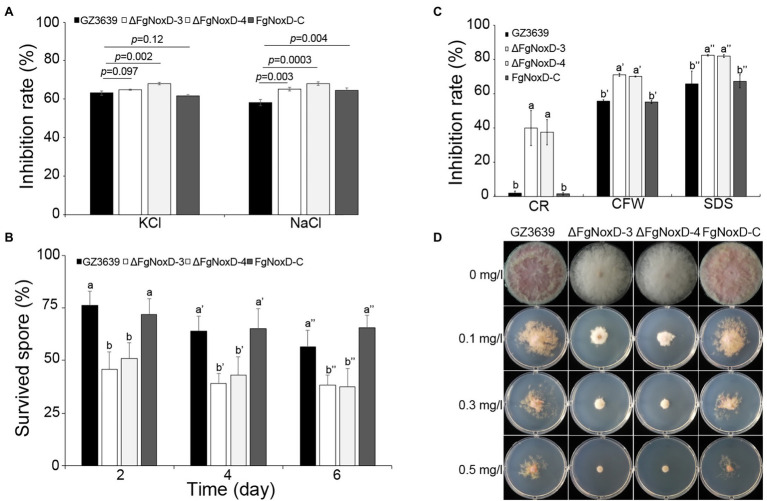
Resistance of GZ3639, ΔFgNoxD, and FgNoxD-C to various stresses. **(A)** Osmotic stress: strain was cultured in MM supplemented with 1.4 M KCl and NaCl for 5 days. Error bars represent the SE from five replicates. **(B)** Cold stress: 10^3^ conidia/ml of each strain were stored at 4°C for 6 days in distilled water. One hundred microliter of each conidia suspension was spread onto PDA and the survived spore was counted after 1 day. Error bars represent the SD from five replicates. **(C)** Cell membrane and cell wall stress test. All strains were cultured in MM without or with 60 mg/L congo red (CR), 50 μg/ml calcofluor white (CFW), 0.01% SDS for 3 days. Error bars represent the SD from five replicates. Values with different letters are significantly different according to Tukey’s test ( *p* < 0.001). **(D)** Fungicide test: the strains were inoculated in MM containing different concentration of prochloraz for 5 days at 25°C. This experiment was repeated five times.

**Figure 4:**
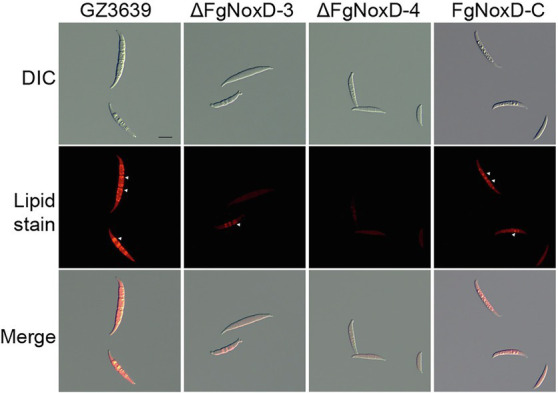
Accumulated lipid body in GZ3639, ΔFgNoxD, and FgNoxD-C. Conidia suspension of GZ3639, ΔFgNoxD, and FgNoxD-C (1 × 10^6^ conidia/ml in distilled water) were store at 4°C for 1 day and stained with Nile Red. White arrow depicts accumulated lipid body. Scale bar = 10 μm.

**Figure 5:**
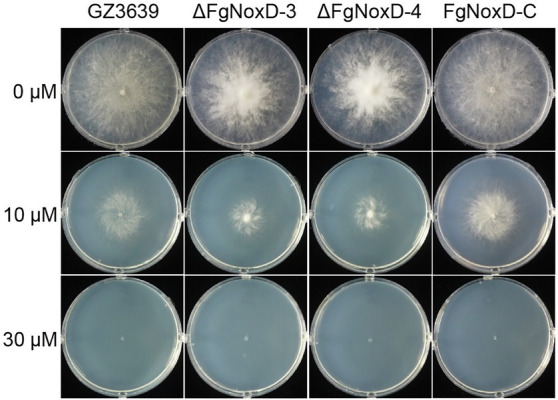
Resistance of GZ3639, ΔFgNoxD, and FgNoxD-C to menadione. *Fusarium graminearum* strain was cultured on MM supplemented with different concentration of menadione for 3 days at 25°C. This experiment was repeated five times.

**Figure 6:**
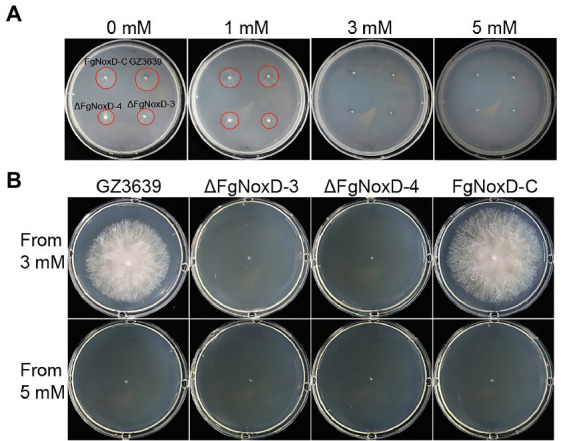
The effect of H_2_O_2_ on survival of GZ3639, ΔFgNoxD, and FgNoxD-C. **(A)** All strains were cultured in MM supplemented with different H_2_O_2_ concentrations for 1 day. **(B)** Strains from media containing 3 or 5 mM H_2_O_2_ were cultured for 3 days. The red circles indicate areas of growing mycelium.

To confirm the role of *FgNoxD* in resistance to various stresses, a series of abiotic stress resistances were tested. ΔFgNoxD showed significantly reduced resistance to osmotic stress when supplied with 1.4 M KCl or 1.4 M NaCl compared to the wild type (Figure 3A). ΔFgNoxD also showed a significantly lower survival rate under cold condition compared to the wild-type and complemented strains (Figure 3B). In cell wall integrity test, ΔFgNoxD showed a significantly reduced inhibition rate compared to the wild type not only in CR supplemented medium but also in CFW supplemented medium. ΔFgNoxD also displayed reduced resistance to SDS, which disrupts cell membrane integrity (Figure 3C). Compared to the wild type, ΔFgNoxD also showed significantly reduced resistance to prochloraz, a fungicide which target the cell membrane (Figure 3D). In addition, the lipid body of ΔFgNoxD was reduced under cold condition (Figure 4). Treatment with menadione showed no significantly difference between deletion mutant and wild type (Figure 5). When H_2_O_2_ was added to the medium, there was no difference between the deletion mutants and the wild type. However, when these strains without mycelium growth (Figure 6A) were transferred to another normal CM, it could be seen that, contrary to the wild type, mycelium growth could not be observed in the deletion mutants, that means H_2_O_2_ was lethal to the deletion mutant (Figure 6).

### *FgNoxD* Is Required for Virulence

**Figure 7:**
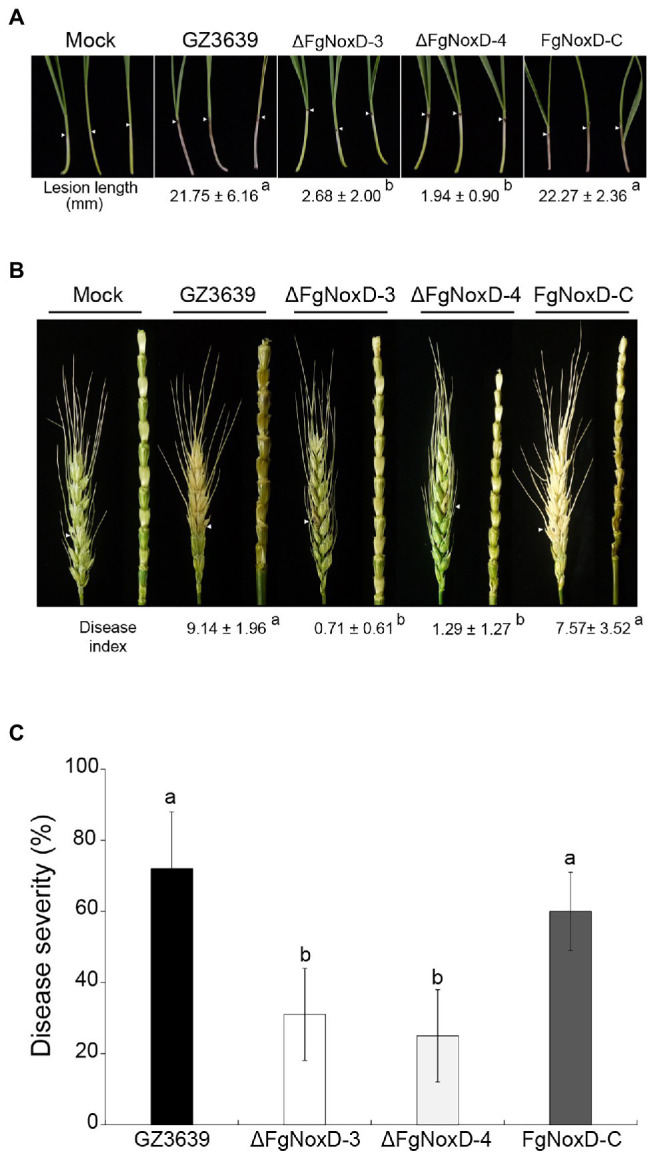
Virulence of GZ3639, ΔFgNoxD, and FgNoxD-C. **(A)** Wheat coleoptiles were inoculated with 2 μl conidial suspension (1 × 10^6^ conidia/ml in 0.01% Tween-20), and lesion length was measured for at least 10 wheat coleoptiles at 10 dpi. This experiment was repeated three times. **(B)** Flowering wheat heads were inoculated with 10 μl conidial suspension (1 × 10^6^ conidia/ml in 0.01% Tween-20) and observed at 14 dpi. Disease index was determined from the number of symptomatic spikelets per wheat head. At least 14 wheat heads inoculated with *F. graminearum* strain were examined in addition to the wheat head rachis. **(C)** Flowering rice heads were dipped into conidial suspension (1 × 10^5^ conidia/ml in 0.01% Tween-20) for 30 s and symptomatic rice grains per rice head were examined at 14 dpi. Disease severity was determined from the number of symptomatic grains per rice head. This experiment was repeated three times. Values with different letters are significantly different according to Tukey’s test ( *p* < 0.001).

The virulence of the deletion mutant was reduced compared with that of the wild type. Compared to the wild type, the lesion length on coleoptile was significantly reduced when coleoptile was inoculated with the deletion mutant (Figure 7A). FHB symptom in wheat heads and rachis inoculated with the deletion mutant were also significantly reduced (Figure 7B). The deletion mutant showed significantly reduced disease severity compared to the wild-type and complemented strains (Figure 7C).

### *FgNoxD* Is Required for DON Production

**Figure 8:**
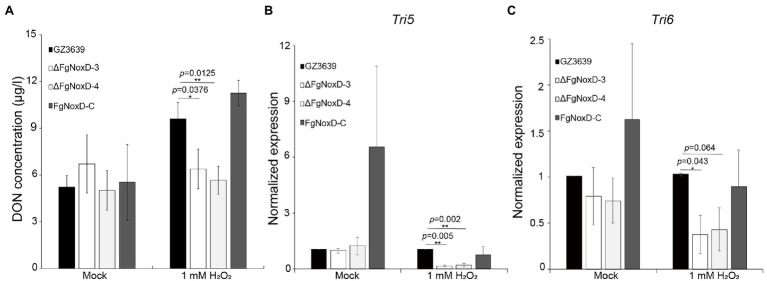
Deoxynivalenol (DON) biosynthesis of GZ3639, ΔFgNoxD, and FgNoxD-C. **(A)** The DON concentration of *F. graminearum* strain. Supernatant of each culture was used to analyze DON concentration. **(B,C)** Relative expression levels of *Tri5* and *Tri6*. GZ3639, ΔFgNoxD and FgNoxD-C were cultivated in GYEP with or without (Mock) 1 mM H_2_O_2_ for 5 days. The mycelia of *F. graminearum* strain was used to measure the transcript expression level. For DON concentration, bars denote SD from three repeated experiments. For qRT-PCR bars denote SE from three repeated experiments with three replications. Asterisks indicate value of *p* (^*^p < 0.05, ^**^p < 0.01) after comparison with *t*-test.

There was no significant difference between the deletion mutant and wild-type strains with respect to DON production when they were cultivated in GYEP medium. However, when the medium was supplemented with H_2_O_2_, the deletion mutant showed reduced DON production compared to the wild type (Figure 8A). The transcript levels of both *Tri5* and *Tri6* showed no significantly differences between ΔFgNoxD and the wild type in GYEP medium but were significantly reduced in ΔFgNoxD compared to the wild type in the medium with H_2_O_2_ (Figures 8B,C).

## Discussion

The multicomponent Nox reduces molecular oxygen to superoxides in a stepwise manner, leading to the production of ROS (Lambeth, 2004). In fungi, these multicomponent enzymes are involved in virulence and differentiation (Malagnac et al., 2004; Egan et al., 2007; Takemoto et al., 2007; Giesbert et al., 2008). Moreover, previous studies in *B. cinerea* revealed that NoxD is involved in vegetative differentiation, colonization of host tissue, and oxidative stress resistance (Siegmund et al., 2015). Similar to previous studies on NoxD, our current study showed that *FgNoxD* in *F. graminearum* is involved in normal vegetative growth, virulence, asexual development, and resistance to various stressors.

Sexual development in *F. graminearum* is a vital factor that leads to its genetic diversity and adaptability in nature (Lee et al., 2011; Ni et al., 2011; Li et al., 2019). Sexual development is also a central strategy to survival in soil or host plant debris in the fields during winter (Guenther and Trail, 2005). Our study showed that ΔFgNoxD completely lost sexual development and also showed reduced resistance to cold stress (Figures 2C, 3B). These results suggest that *FgNoxD* plays an important role in *F. graminearum* survival during winter. The accumulated of lipid bodies act as reserves for perithecium development (Guenther et al., 2009; Son et al., 2011). Moreover, lipids are known to be involved in cold tolerance and survival in fungi (Istokovics et al., 1998). Our data also showed that lipid accumulation in ΔFgNoxD was reduced compared to the wild-type strain (Figure 4), which might have resulted in the abolishment of sexual development and reduction in cold stress resistance.

The fungal cell wall is an essential component with great plasticity that plays a vital role in normal cell growth and protection of cells from osmotic stress (Gow et al., 2017; Garcia-Rubio et al., 2019). ΔFgNoxD showed reduced vegetative and aerial hyphae growth (Figures 2A,B) and reduced resistance to osmotic stress compared to the wild type (Figure 3A). ΔFgNoxD was more sensitive to cell wall perturbing factor compared to the wild-type and complemented strains (Figure 3C), leading to the defects in vegetative growth and osmotic stress in the mutant. In addition, these results showed that *FgNoxD* plays a pivotal role in cell wall integrity in *F. graminearum*.

The fungal cell membrane is also an important component that is enriched with diverse lipids, such as sphingolipids and sterols (Sant et al., 2016). These lipids regulate fungal pathogenicity through lipid–protein and lipid–lipid interactions (Rella et al., 2016). In our study, ΔFgNoxD showed significantly reduced fungicide resistance compared to the wild type when the medium was supplemented with prochloraz, a fungicide that targets the fungal cell membrane. On the other hand, cell membrane also involved in response to osmotic stress (You et al., 2012; Freitag et al., 2014; Ren et al., 2019). The results showed that ΔFgNoxD was more sensitive to osmotic stress as well as cell membrane inhibitors than the wild-type and complemented strains (Figures 3A,C). Furthermore, lipid accumulation in ΔFgNoxD was significantly reduced (Figure 4). These results suggested that *FgNoxD* plays an important role for cell membrane integrity, and is tightly linked to virulence in *F. graminearum* (Figure 7).

The virulence of *F. graminearum* against the host plant can be ascribed to many factors, including resistance to ROS produced by the host plant and biosynthesis of trichothecenes (Boenisch and Schafer, 2011; Barna et al., 2012; Mentges and Bormann, 2015). ROS is a common by-product of both eukaryotic and prokaryotic organisms (Aguirre et al., 2005). ROS has a well-established damaging effect on cell components and are commonly used in plant defense systems (Jones and Dangl, 2006; Halliwell and Gutteridge, 2015). When plants recognize a pathogen, plant cells are capable of producing a burst of ROS, initially comprising H_2_O_2_, which can react with the proteins, DNA, and lipids of the pathogen to accelerate cell death (Sharma and Davis, 1997; O’Brien et al., 2012). Therefore, fungi must deactivate ROS produced by plants for successful plant infections. In our study, ΔFgNoxD showed reduced resistance to oxidative stress compared to the wild-type and complemented strains (Figure 6). The production of DON, which is an important virulence factor in *F. graminearum*, is triggered by H_2_O_2_ (Proctor et al., 1995; Audenaert et al., 2010). The amount of DON production triggered by H_2_O_2_ was reduced in ΔFgNoxD compared to the wild type (Figure 8A). Meanwhile, when exposed to H_2_O_2_, the expression levels of *Tri5* and *Tri6* were all significantly decreased in ΔFgNoxD compared to the wild-type and complemented strains (Figures 8B,C). In summary, the reduced virulence of ΔFgNoxD in host plants may be a result of reduced resistance to oxidative stress and DON biosynthesis.

In this study, we identified that *FgNoxD* plays an important role in the virulence of *F. graminearum*. The loss of virulence in ΔFgNoxD could be due to reduced mycelia growth, cell wall and membrane integrity, and resistance to ROS. *FgNoxD* contributed to the spread of the infected *F. graminearum* throughout the entire spike (Figure 7B). In addition, *FgNoxD* also plays an important role in sexual development and conidial production (Table 2). Therefore, understanding the role of *FgNoxD* may provide a new way to control FHB in the field. This study expands our knowledge of the Nox family in *F. graminearum*, and future studies will allow further dissection the role of *FgNoxD* and interaction with other Nox family members in *F. graminearum*.

## Data Availability Statement

The original contributions presented in the study are included in the article/Supplementary Material; further inquiries can be directed to the corresponding author.

## Author Contributions

TL and DK designed the experiments. DK created figures and tables. TL, DK, and JL wrote the manuscript. All authors contributed to the article and approved the submitted version.

## Funding

This work was supported by the National Research Foundation (2020R1A2C2013617), LG Yonam Foundation of Korea, and Green Fusion Technology Program funded by Ministry of Environment, Republic of Korea.

## Conflict of Interest

The authors declare that the research was conducted in the absence of any commercial or financial relationships that could be construed as a potential conflict of interest.

## Footnotes

^1^ https://www.ncbi.nlm.nih.gov
