# Supplementary material for: NADPH Oxidase Gene, FgNoxD, Plays a Critical Role in Development and Virulence in Fusarium graminearum
Source: Front Microbiol. 2022 Mar 3;13:822682. doi: 10.3389/fmicb.2022.822682 (PMC8928025; doi:10.3389/fmicb.2022.822682)
Supplement: Supplementary file 1 [file Data_Sheet_1.docx]

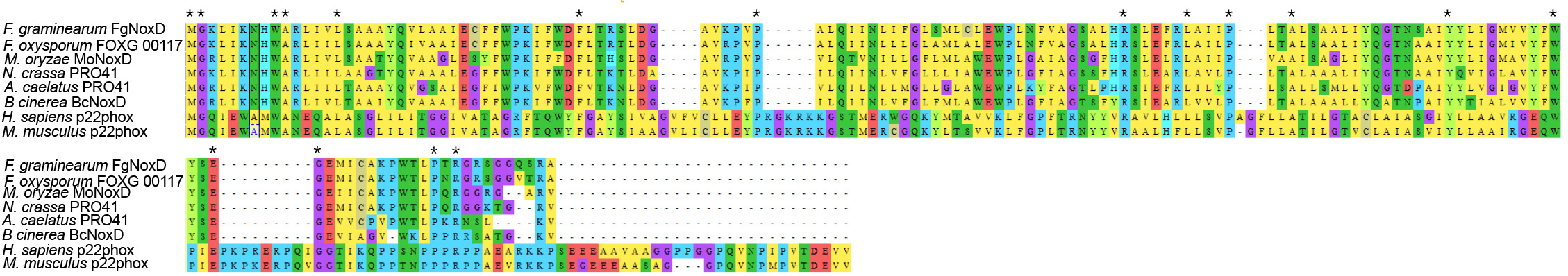


**Supplementary Figure 1.** Alignment of NoxD homologues. All amino acid sequence aligned using MEGA X (version 6.0).
